# Modeling of Rifampicin-Induced CYP3A4 Activation Dynamics for the Prediction of Clinical Drug-Drug Interactions from *In Vitro* Data

**DOI:** 10.1371/journal.pone.0070330

**Published:** 2013-09-24

**Authors:** Fumiyoshi Yamashita, Yukako Sasa, Shuya Yoshida, Akihiro Hisaka, Yoshiyuki Asai, Hiroaki Kitano, Mitsuru Hashida, Hiroshi Suzuki

**Affiliations:** 1 Department of Drug Delivery Research, Graduate School of Pharmaceutical Sciences, Kyoto University, Kyoto, Japan; 2 Department of Pharmacology and Pharmacokinetics, The University of Tokyo Hospital, Faculty of Medicine, The University of Tokyo, Tokyo, Japan; 3 Open Biology Unit, Okinawa Institute of Science and Technology Graduate University, Okinawa, Japan; 4 Sony Computer Science Laboratories, Inc, Tokyo, Japan; 5 Institute for Integrated Cell-Material Sciences, Kyoto University, Kyoto, Japan; 6 Department of Pharmacy, The University of Tokyo Hospital, Faculty of Medicine, The University of Tokyo, Tokyo, Japan; UC Davis School of Medicine, United States of America

## Abstract

Induction of cytochrome P450 3A4 (CYP3A4) expression is often implicated in clinically relevant drug-drug interactions (DDI), as metabolism catalyzed by this enzyme is the dominant route of elimination for many drugs. Although several DDI models have been proposed, none have comprehensively considered the effects of enzyme transcription/translation dynamics on induction-based DDI. Rifampicin is a well-known CYP3A4 inducer, and is commonly used as a positive control for evaluating the CYP3A4 induction potential of test compounds. Herein, we report the compilation of *in vitro* induction data for CYP3A4 by rifampicin in human hepatocytes, and the transcription/translation model developed for this enzyme using an extended least squares method that can account for inherent inter-individual variability. We also developed physiologically based pharmacokinetic (PBPK) models for the CYP3A4 inducer and CYP3A4 substrates. Finally, we demonstrated that rifampicin-induced DDI can be predicted with reasonable accuracy, and that a static model can be used to simulate DDI once the blood concentration of the inducer reaches a steady state following repeated dosing. This dynamic PBPK-based DDI model was implemented on a new multi-hierarchical physiology simulation platform named PhysioDesigner.

## Introduction

Cytochrome P450 enzymes (CYPs) are implicated in many clinically relevant drug-drug interactions (DDI), as the metabolism reactions catalyzed by this enzyme family are the dominant route of elimination for the majority of drugs. Inhibition of the CYPs can lead to an unwanted elevation in the blood level of drugs administered concomitantly, which can result in life-threatening adverse drug reactions [1], [2]. Induction of CYP expression is not normally considered to be a safety concern, but can lead to inadequate drug efficacy [3]. For example, co-administration of rifampicin and cyclosporine results in excess metabolism of cyclosporine leading to allograft rejection in transplanted patients [4]–[6]. Thus, predictions of *in vivo* DDIs from *in vitro* metabolism data are becoming increasingly important during the process of preclinical drug development.

Various mathematical models have been proposed to predict potential clinical drug-drug interactions from *in vitro* data [7]–[10]. However, induction studies are generally more difficult to conduct compared with inhibition studies, as they need a cell-based system that allows evaluation of gene transcription and protein expression. The simplest model is one in which a static score of degree of induction is calculated from the average plasma concentration of an inducer using *in vitro EC_50_* and *E_max_* estimates [11], [12]. The potential for induction-based DDI for any particular drug combination is then predicted based on the proportion of the drugs' total body clearance attributable to the enzymes induced. Dynamic models consider fluctuations in the levels of enzyme activity [13]–[15]. The clearance rate of substrate drugs can be dynamically altered by the acceleration of enzyme synthesis in an inducer concentration-dependent manner. A recent study indicated that a dynamic model, although not a marked improvement over the static model, tended to give better predictions for the 50 clinical DDI cases studied [16].

To date, the dynamic models reported are all indirect pharmacokinetic/pharmacodynamic (PK/PD) models [13]–[15] which assume that an inducer accelerates the enzyme synthesis in a concentration-dependent manner. Since enzyme synthesis is assumed to obey zero-order kinetics, the action of the inducer on enzyme synthesis starts immediately. Therefore, the gradual increase in CYP activity over several days' exposure to the inducer is attributed simply to the slow degradation rate of these enzymes. However, several studies have indicated that it takes at least a few days for the mRNA to reach a maximum level [17], [18]. To evaluate the kinetics of enzyme induction, it is important to consider the time courses of sequential mRNA and enzyme synthesis.

The present study is aimed at developing a hybrid simulation model for predicting the dynamics of induction-based DDI, in which a whole-body physiologically based pharmacokinetic (PBPK) model and an enzyme transcription/translation dynamics model are implemented. Feasibility of this hybrid model was investigated using rifampicin, a well-characterized and potent inducer of CYP3A4. Rifampicin is frequently used as a positive control or calibrator for evaluating the CYP3A4 induction potential of test compounds. Therefore, a large amount of *in vitro* rifampicin data is available in the literature. In general, cultures of primary human hepatocytes are believed to be the best *in vitro* model for simulating *in vivo* conditions. However, considerable functional variability of donor hepatocytes has been observed [19], [20]. To obtain non-biased parameters regarding transcription and translation of CYP3A4, we systematically collected *in vitro* data and analyzed them using an extended least squares method [21], [22] that allows the estimation of kinetic parameters while taking inter- and intra-individual variability into account. Using the parameters estimated from *in vitro* human hepatocytes, we then predicted clinical pharmacokinetics of CYP3A4 substrate drugs in the presence of concomitantly administered rifampicin.

## Materials and Methods

### Data Collection

The *fm_CYP3A4_* values, i.e., the apparent contribution of CYP3A4 to drug oral clearance, were obtained for 15 CYP3A4 substrate drugs in a previous report [23], [24]. These values were estimated from the increase in *AUC_oral_* of the drugs tested resulting from the action of CYP3A4 inhibitors, as observed in 53 separate clinical DDI studies [23]. *AUC_oral_* is the area under the blood concentration-time profile following oral administration. Information on the pharmacokinetics of CYP3A4 substrate drugs when co-administered with rifampicin (see Table S1) was also obtained from the literature [25]–[40]. The dosage regimen of oral rifampicin was also considered in the present analysis. Clinical pharmacokinetic data of rifampicin with different oral dosage regimens were obtained from a report by Acocella et al. [41]. In addition, *in vitro* rifampicin induction data of CYP3A4 mRNA expression and/or enzyme activity in primary cultures of human hepatocytes were also collected [17], [42]–[46].

### Modeling of the induction dynamics of CYP3A4 expression in human hepatocytes

Following the onset of treatment of hepatocytes with rifampicin, expression of CYP3A4 mRNA was up-regulated after an initial time delay, and reached maximum level on day 2 [17]. Taking into account that rifampicin induces expression of CYP3A4 via activation of the pregnane X receptor (PXR), a dynamic model with a putative receptor was defined using the following equations:
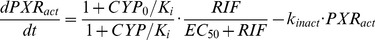
(1)

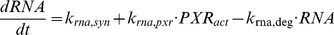
(2)

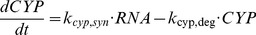
(3)where *RIF*, *PXR_act_*, *RNA*, and *CYP* are the concentration of rifampicin, normalized amount of activated PXR, CYP3A4 mRNA level, and CYP3A4 enzyme level, respectively, *CYP_0_*, *EC_50_*, *K_i_*, and *k_inact_* are the baseline level of CYP3A4 enzyme, concentration of rifampicin at half-maximum PXR activation, the constant for negative feedback inhibition, and the inactivation rate constant for activated PXR, respectively. *k_rna,syn_* and *k_rna,pxr_* are rate constants for baseline and PXR-mediated synthesis of CYP3A4 mRNA, and *k_rna,deg_* is the rate constant for its sequestration, while *k_cyp,syn_* and *k_cyp,deg_* are rate constants for the synthesis and sequestration of CYP3A4 enzyme. In the model, a delay in the early phase of CYP3A4 mRNA expression after addition of the inducer was assumed to be attributable to the time required for activation of PXR, while the accelerated decay of this mRNA was thought to result from the subsequent negative feedback inhibition by PXR according to CYP3A4 level. In general, induction of mRNA and subsequent CYP3A4 enzyme levels is evaluated as the fold increase over the value observed on day 0. If the levels of mRNA and enzyme return to their original values (*RNA_0_* and *CYP_0_*, respectively) by the removal of a stimulus, the following relationships should be satisfied:



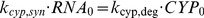
(4)

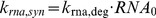
(5)


Therefore, using 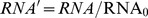 and 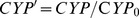, Eqs. 1–3 can be replaced with:
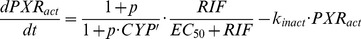
(6)

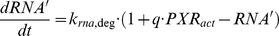
(7)

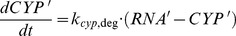
(8)where



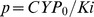
(9)

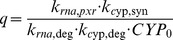
(10)


Since the considerable inter-donor variability of drug metabolism by human hepatocytes has been attributed to variations in the baseline level of the CYP3A4 activity present [20], an extended least square analysis was performed by considering the effect of inter-donor variability on *CYP_0_* (that is, *p* and *q*). This analysis was carried out using the ADVAN9 routine in NONMEM 7.2 (Icon, Inc., Dublin, Ireland).

### Conventional modeling of the induction dynamics of CYP3A4 activity

An indirect effect model for enzyme induction [13]–[15] can be represented as follows:
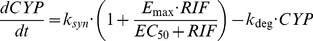
(11)where *k_syn_* and *k_deg_* are rate constants for baseline synthesis and sequestration of CYP3A4 enzyme, respectively. Assuming that the level of enzyme prior to administration of rifampicin (*CYP_0_*) was under the steady state, the following relationship should be satisfied:



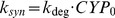
(12)Replacing the *k_syn_* of Eq. 11 and normalizing it with *CYP_0_* (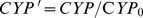), we obtain:
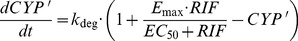
(13)


An extended least square analysis was performed by considering the effect of inter-donor variability on *E_max_*. This analysis was carried out using the ADVAN9 routine in NONMEM 7.2.

### Analysis of CYP3A4 activity induction by a simple static model

Using only 72-h data, the *E_max_* and *EC_50_* values for induction of CYP3A4 by rifampicin were estimated from the following equation:
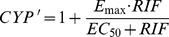
(14)


An extended least square analysis was performed by considering the effect of inter-donor variability on *E_max_*, similarly to the case of the indirect effect model mentioned before.

### Modeling of the clinical pharmacokinetics of rifampicin following repeated oral dosing

PBPK models are mechanistically rigorous models that incorporate anatomical and biochemical information into descriptions of pharmacokinetics. To construct PBPK models, measurements of drug concentrations in each organ and tissue are required. However, only blood and urine data are generally available in clinic. As an intermediate approach, a PBPK model which gives an abstracted blood compartment and considers only recirculation between blood and liver has been utilized [7]. It has been demonstrated that the simplified PBPK model allows *in vitro*-*in vivo* extrapolation of hepatic drug metabolism [7]. Rifampicin clearance is known to be a nonlinear saturable process that accelerates during repeated oral dosing [41]. The simplified PBPK model was modified taking this specialized aspect of rifampicin pharmacokinetics into consideration. The mass-balance equations were:
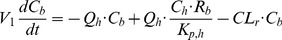
(15)

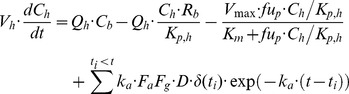
(16)

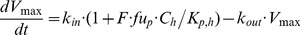
(17)where *C_b_* and *C_h_* are concentrations of the drug in the blood and liver, respectively, and *V_max_* is the inducible maximum hepatic elimination rate. *V_1_*, *CL_r_*, *Q_h_*, *R_b_*, and *K_p,h_* are the volume of systemic circulation, renal clearance, hepatic plasma flow rate, blood/plasma distribution ratio, and liver/plasma distribution ratio, respectively. *V_h_*, *fu_p_*, *K_m_*, *k_a_*, *F_a_F_g_*, and *D* are the volume of liver, fraction unbound in plasma, Michaelis-Menten constant for hepatic elimination, absorption rate constant, product of fraction absorbed and intestinal availability, and amount of oral dose, respectively. *k_in_*, *k_out_*, and *F* are the rate constant for synthesis of hepatic elimination activity, rate constant for decay of hepatic elimination activity, and coefficient for auto-induction, respectively. *CL_r_* and *fu_p_* were assumed to be 1.8 L/h and 0.2 [47], respectively. *Q_h_* and *V_h_* were assumed to be 96.6 L/h and 1.4 L, respectively [7]. Assuming that *F_a_F_g_* and *R_b_* were both at unity, *V_1_*, *K_m_*, *k_a_*, *K_p,h_*, *k_in_*, *k_out_*, and *F* were estimated by curve-fitting to blood concentration-time profiles following repeated oral dosing of rifampicin with different doses [41]. The parameter estimation procedure was carried out with the ADVAN9 routine in NONMEM 7.2.

### Simulation of drug-drug interactions with rifampicin

In the case of drugs that are mostly metabolized by the liver, induction-based DDI occurring after oral administration is represented by:
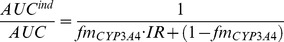
(18)where *AUC* and *AUC^ind^* are areas under the blood concentration profile in the absence and presence of an inducer, respectively. *fm_CYP3A4_* and *IR* are the fraction of the drug metabolized by CYP3A4 and the relative activity of CYP3A4 induced by the inducer, respectively. This equation has been derived using the following assumptions: the substrate drug is eliminated solely by the liver, and the induction of CYP3A4 in the intestine is negligible. The *fm_CYP3A4_* values for each substrate drug were obtained from the literature [23], [24]. In the previous article [24], 53 induction-based DDI data sets in human were collected and compiled without any normalization, demonstrating that the degree of DDIs could be comprehensively explained by the *IR* values of various inducers determined from *in vivo* data by taking simvastatin as a standard CYP3A4 substrate. In the present study, only data for rifampicin were taken from the compiled data. The *IR* value for rifampicin was estimated using *in vitro* parameters with the following process: Using Eqs. 15–17, the unbound concentration of rifampicin in the liver (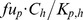) was computed. By substituting it for the variable *RIF* in Eq. 6 or 13, a time-course for the degree of induction of CYP3A4 (*CYP*') *in vivo* was estimated by Eqs. 6–8 or Eq. 13. The *IR* was defined as the average of *CYP*' over the interval.

To simulate the blood concentration-time profile of a CYP3A4 substrate drug in the presence of rifampicin, a PBPK model for the substrate, similar to that for rifampicin (Eqs. 15–17), was considered. Assuming that hepatic elimination is linear, the mass-balance equation for the liver was replaced with:
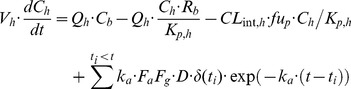
(19)where *CL_int,h_* depicts intrinsic clearance for the substrate. Pharmacokinetic parameters for CYP3A4 substrates were obtained by curve-fitting to their blood concentration profiles as has been reported previously [7]. For simulation of DDI with rifampicin, *CL_int,h_* was assumed to be dependent on *CYP*':
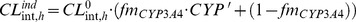
(20)where 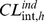 and 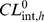 are intrinsic clearances for the substrate in the presence and absence of rifampicin, respectively. Thus, two PBPK models for the inducer and substrate were bridged with the CYP3A4 induction dynamics model to compute the DDI with rifampicin.

### PHML-SBML hybrid simulation

Physiological Hierarchy Markup Language (PHML) is a markup language that can explicitly describe the multi-level hierarchical structures of physiological functions in mathematical models. One of the remarkable features of PHML is that it enables the embedding of Systems Biology Markup Language (SBML) [48] models as a module. To make a DDI model more readable and reusable, two PBPK models for both inducer and substrate were stored in the PHML format, and connected to each other via a functional module representing subcellular enzyme induction, of which the contents were implemented in SBML. The PHML and SBML models were developed using open source modeling platforms, PhysioDesigner (formerly *insilico*IDE) and CellDesigner, respectively [49], [50]. PhysioDesigner and CellDesigner are freely available at http://physiodesigner.org and http://celldesigner.org.

**Figure 1:**
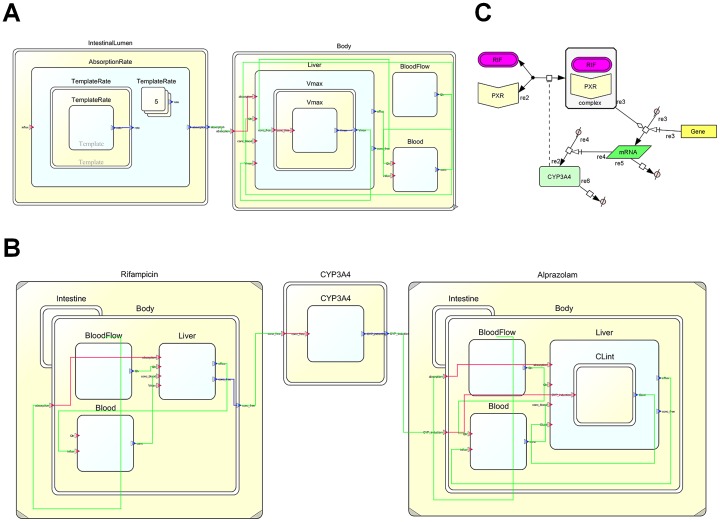
Snapshots of DDI models implemented in multi-hierarchical physiology simulation platforms. Fig./translation dynamics model for CYP3A4 following administration of the drug, as implemented on CellDesigner. Fig. 1C represents a PBPK-based DDI model, where the enzyme induction model was hybridized. Yellow and white rectangles represent the capsule module and functional module, respectively. Modules can communicate by connecting their ports with an edge.

Fig. 1 represents snapshots of the DDI model implemented in the simulation platform. As shown in Fig. 1A, a PBPK model is primarily composed of two modules corresponding to the intestinal lumen and body. Between these modules, it is enough to pass only the value of an intestinal drug absorption rate. To ensure maintainability and scalability of the model, these modules were capsuled to hide unnecessary values, and opened with only a port to pass the absorption rate value. By connecting the ports with an edge, these capsule modules can communicate with each other. Each module was further modeled in a hierarchical manner. Using a template/instance framework of PHML, the absorption rate was calculated in the intestinal lumen module by summing up the values from each of the instances corresponding to multiple doses. The body module includes the functional modules for the liver and blood, in addition to a module for common static variables. Differential equations and variables were implemented in the liver and blood modules. Upon developing PBPK models for an inducer and a CYP3A4 substrate drug, the models were bridged with a capsuled functional module for induction of CYP3A4 (Fig. 1B). The CYP3A4 induction module receives the unbound concentration of the inducer in the liver from the inducer PBPK model and provides the *IR* value for the substrate PBPK model. However, the module was simply a frame, and its object was implemented in a SBML format. Fig. 1C represents a SBML model for induction of CYP3A4 developed using CellDesigner.

## Results

### Modeling of CYP induction dynamics

**Figure 2:**
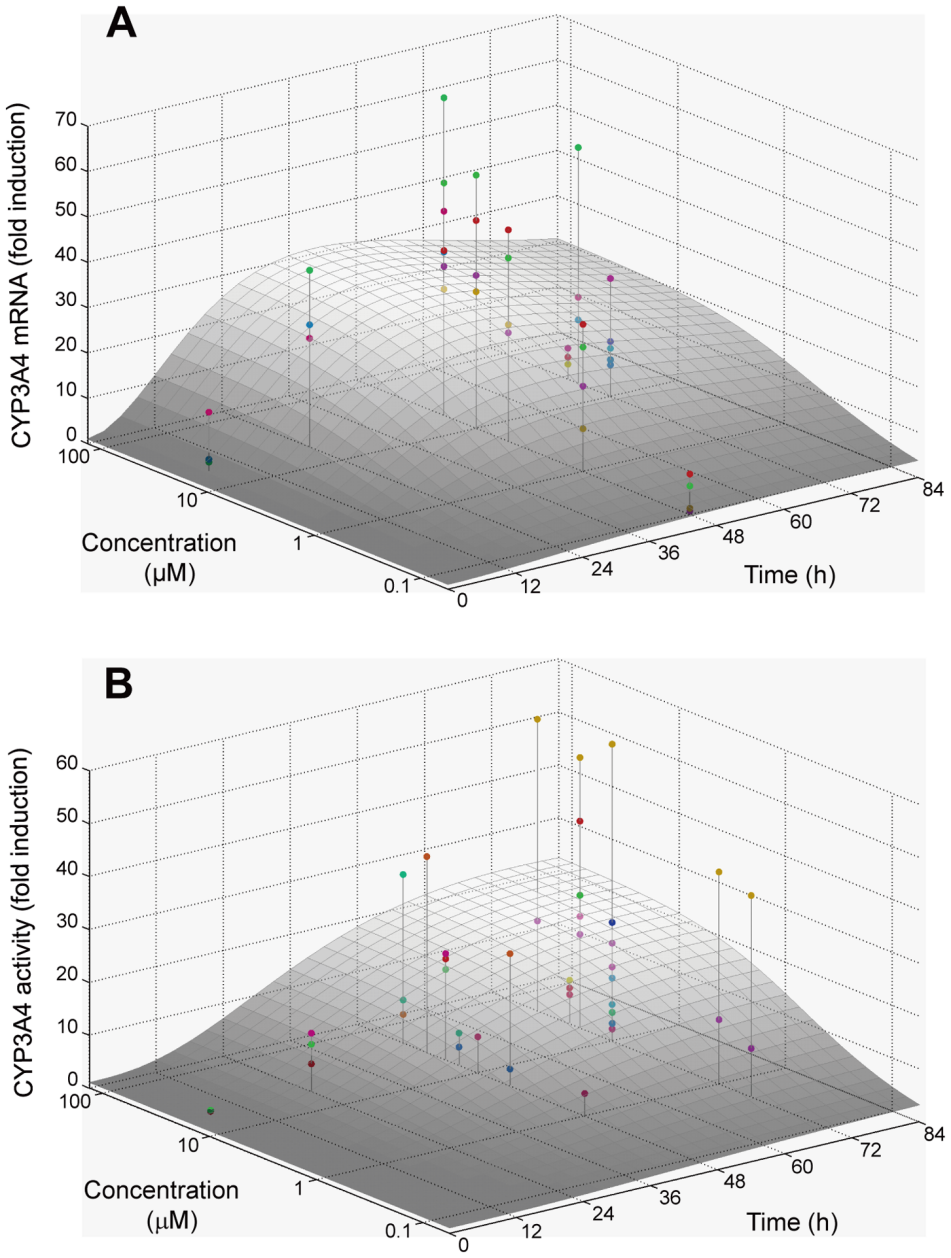
Curve-fitting to experimental data of the induction of CYP3A4 by rifampicin in human hepatocytes. Fig.-normalized data and corresponding equations, i.e., Equations 6–8, were used for this analysis, assuming that inter-individual variability for induction is because of differences in baseline CYP3A4 activity. The surface curves represent the averages.

The pooled data set obtained from 24 different sources comprised 43 and 40 data points for CYP3A4 enzyme activity and mRNA expression levels, respectively. Considering the effect of inter-donor variability on the baseline level of CYP3A4 activity, an extended least square analysis was performed based on Eqs. 6–8 (see Methods). The parameters *EC_50_*, *k_inact_*, *k_rna,deg_*, *k_cyp,deg_*, *p* and *q* were estimated to be 1.18 µM, 0.0530 h^–1^, 0.0282 h^–1^, 0.313, and 4.34, respectively, in addition to the inter-donor variability of *CYP_0_* (ω^2^) of 0.318. Interestingly, the *k_cyp,deg_* estimated was comparable to the one that was previously optimized for better *in vitro*/*in vivo* extrapolation (0.03 h^–1^) [51], [52]. Fig. 2 represents simulated surface plots for mean CYP3A4 activity and mRNA expression as a function of concentration and time. Expression of mRNA reached a maximum level at ∼40 h following the onset of incubation with rifampicin, whereas the peak of CYP3A4 activity induction was delayed in comparison.

The data set for induction of CYP3A4 activity was also analyzed based on a conventionally used indirect effect model. However, simultaneous estimation of all parameters by curve fitting failed, probably because estimation of *k_deg_* requires a clear observation of the maximally induced state in the profile. Alternatively, using the *k_deg_* value from the literature [51], [52], the *EC_50_* and *E_max_* values were estimated by curve-fitting. When the *k_deg_* value was a default value of the Symcyp simulator (0.0072 h^–1^), the *EC_50_* and *E_max_* values were estimated to be 0.283 µM and 37.1, respectively, in addition to a ω_Emax_^2^ of 0.726. When the *k_deg_* corrected for more accurate *in vitro*-*in vivo* extrapolation (0.03 h^–1^) [51], [52] was used, the *EC_50_* and *E_max_* values were estimated to be 0.269 µM and 16.7, respectively, in addition to a ω_Emax_^2^ of 0.702.

When the analysis based on a simple static model was performed using only 72-h data, the *EC_50_* and *E_max_* values were estimated to be 0.281 µM and 14.8, respectively. The ω_Emax_^2^ value was 0.874.

### Modeling of the clinical pharmacokinetics of rifampicin

**Figure 3:**
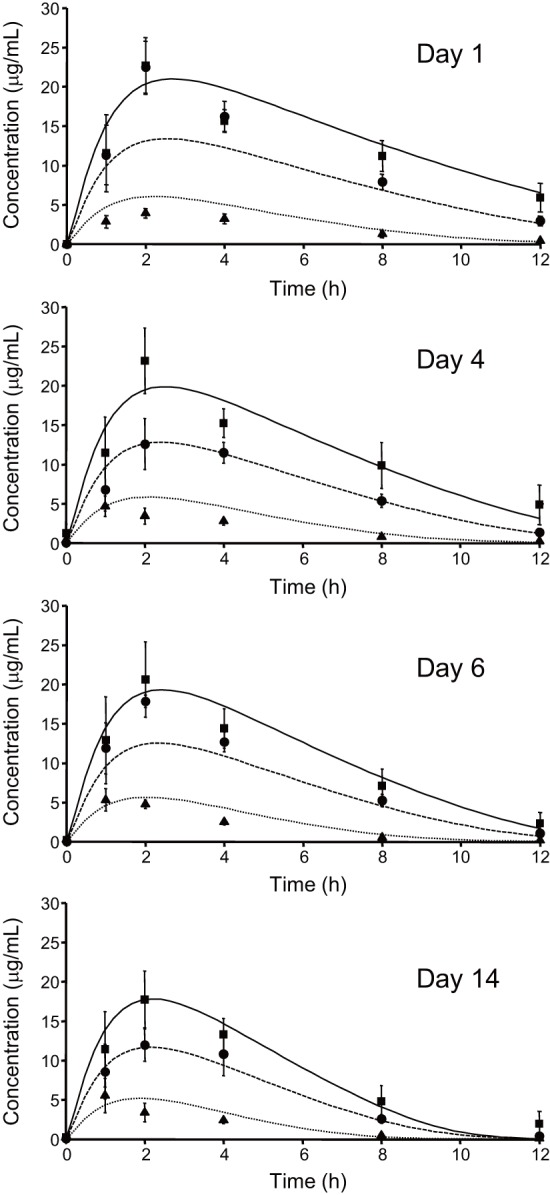
Nonlinear curve-fitting to the blood concentration of rifampicin with repeated oral administration. Clinical data measured on day(Ref. 40) were simultaneously analyzed based on a PBPK model considering an auto-inducible metabolic process (Eqs. 15–17). Theoretical curves are represented for each data set. Keys: 300 mg, b.i.d. (▴, dotted line); 600 mg, q.d. (•, broken line); 900 mg q.d. (▪, solid line).

**Figure 4:**
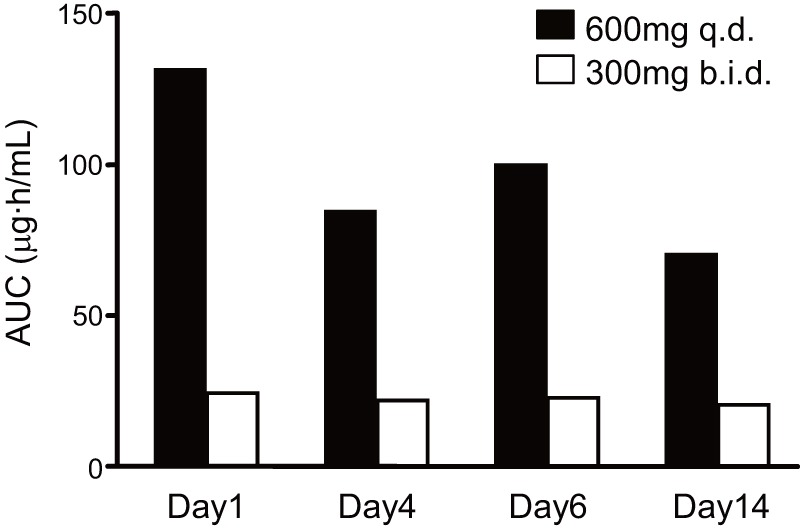
*AUC* measurements of the blood concentration-time profile for oral rifampicin with different dosage regimens. The *AUC* values were calculated from clinical data (Ref. 40) using a trapezoidal method. Note that 600 mg q.d. and 300 mg b.i.d. are the same in terms of total daily dose.

Blood concentration-time profiles following repeated oral administration of rifampicin were simultaneously analyzed to estimate its pharmacokinetic parameters based on a simplified PBPK model. The estimated *k_a_*, *V_1_*, *K_m_*, *K_p,h_*, *k_in_*, *k_out_*, and *F* values were 0.963 h^–1^, 17.2 L, 0.370 mg/L, 10.6, 0.0193 mg/h, 5.75×10^–4^ h^–1^, and 8.64 L/mg, respectively. Fig. 3 represents simulation curves for the blood concentration of rifampicin when using different oral doses, together with experimentally obtained values. To confirm the nonlinearity of rifampicin pharmacokinetics, *AUC_0–12h_* for 300 mg b.i.d. (twice a day) and 600 mg q.d. (once a day) were calculated (Fig. 4). Even though the total daily dose is the same, the *AUC_0–12h_* for 300 mg b.i.d. rifampicin was much smaller than that for 600 mg q.d. This result could be successfully explained by considering it to be a saturable elimination process. Auto-inducible elimination of rifampicin was described by a concentration-dependent increase in *V_max_*.

### *In vitro-in vivo* extrapolation

**Figure 5:**
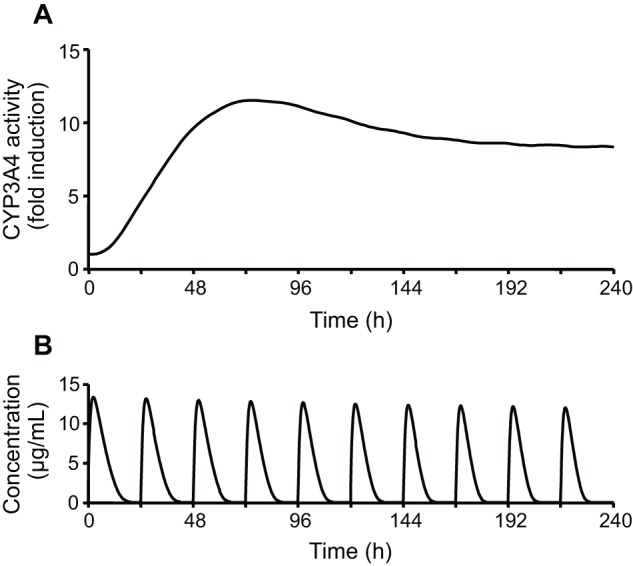
Simulation of the induction of CYP3A4 following repeated oral dosing of rifampicin. Fig.Equations 6–8 and 15–17 were used for this simulation.

Using Eqs. 15–17, the concentration of unbound rifampicin in the liver was computed. The profile was convoluted into Eq. 6 to estimate CYP3A4 induction under clinical conditions, assuming that the mechanism of CYP3A4 induction is equivalent between *in vitro* and *in vivo* states. Fig. 5 shows a simulation of CYP3A4 induction following repeated oral dosing of rifampicin. The level of CYP3A4 activity was transiently increased, peaking on day 4, and then stabilizing on day 6 or later. Fluctuation of CYP3A4 activity arising from repeated dosing of rifampicin was minimal, unlike that of the blood concentration of the drug. Therefore, a static model for enzyme induction would be sufficient to describe the DDI occurring after rifampicin has been repeatedly administered for more than 5 days.

**Figure 6:**
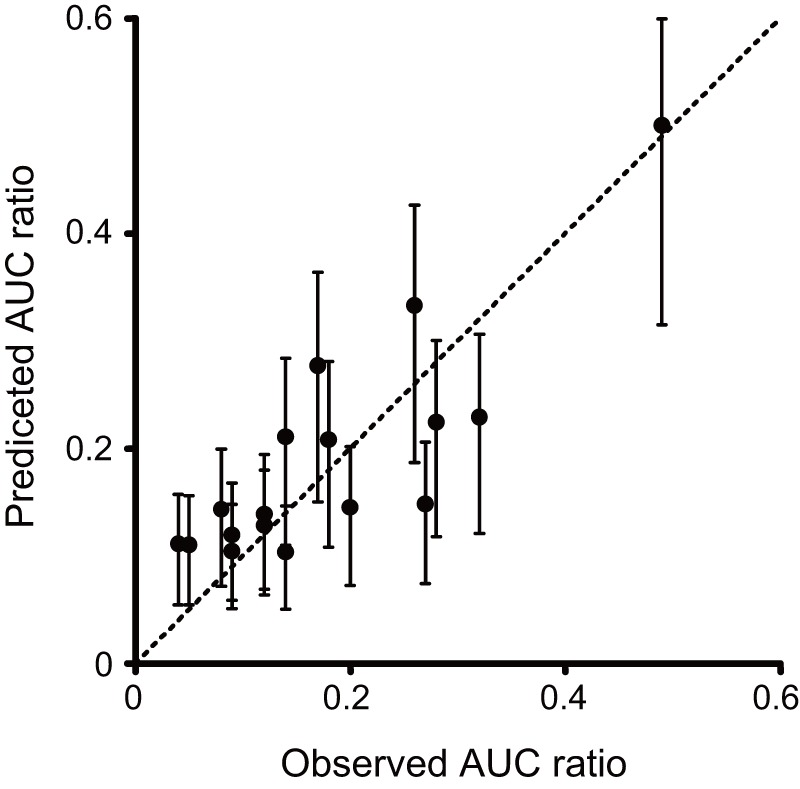
Correlation between predicted and observed *AUC* values for various drugs co-administered with rifampicin. This figure was produced using the values listed in Table 1. Error bars for predicted values represent the standard deviation from the inter-individual variability in baseline CYP3A4 activity. Not that this variability was estimated using extended least squares analysis of *in vitro* data.

Table 1 summarizes clinical DDI results between rifampicin and drugs known to be metabolized by CYP3A4. The *IR* values were estimated as an average of CYP3A4 activity induction for the day studied, according to the dose, dosing interval, and number of days treated with rifampicin. Using *IR* and *fm_CYP3A4_* for each drug, reduction of *AUC* because of co-administration of rifampicin was calculated and compared with clinical data (Fig. 6). The predictive correlation coefficient (*Q* ^2^) and standard deviation of prediction errors (*SDEP*) were 0.684 and 0.0630, respectively. Thus, the reduction of *AUC* for various drugs was predicted with fairly good accuracy when using *in vitro* parameters for CYP3A4 induction.

**Table 1:** Prediction of DDIs for various CYP3A4 substrate drugs with concomitantly administered rifampicin.

Substrate		clinical DDI^b)^				predicted DDI^c)^	
name	fm_CYP3A4_^a)^	daily dose of rifampicin (mg)	days	AUC ratio	Ref. ID	induction ratio (IR) of CYP3A4 activity	AUC ratio
alprazolam	0.75	450	4	0.12	35	9.25 (6.51–19.0)	0.14
atorvastatin	0.68	600	5	0.20	25	9.64 (6.81–19.7)	0.15
buspirone	0.99	600	5	0.088	28	9.64 (6.81–19.7)	0.10
cyclosporine	0.80	600	11	0.27	36	8.16 (5.81–16.5)	0.15
gefitinib	0.39	600	16	0.17	31	7.68 (5.48–15.5)	0.28
imatinib	0.28	600	11	0.26	26	8.16 (5.81–16.5)	0.33
mefloquine	0.44	600	7	0.32	38	8.63 (6.14–17.5)	0.23
midazolam	0.92	600	5	0.041	40	9.64 (6.81–19.7)	0.11
midazolam	0.92	600	9	0.12	27	8.36 (5.94–16.9)	0.13
nifedipine	0.78	600	7	0.082	37	8.63 (6.14–17.5)	0.14
prednisolone	0.18	480	30	0.49	30	6.55 (4.71–13.1)	0.50
simvastatin	1.00	600	9	0.090	27	8.36 (5.94–16.9)	0.12
simvastatin	1.00	600	5	0.14	29	9.64 (6.81–19.7)	0.10
telithromycin	0.49	600	7	0.14	39	8.63 (6.14–17.5)	0.21
triazolam	0.93	600	5	0.051	34	9.64 (6.81–19.7)	0.11
zolpidem	0.40	600	5	0.28	33	9.64 (6.81–19.7)	0.22
zopiclone	0.44	600	5	0.18	32	9.64 (6.81–19.7)	0.21

a) Fraction of the drug metabolized by CYP3A4 (fmCYP3A4) and clinical DDI data were taken from the article of Ohno et al. [24].

b) Clinical data were obtained from the articles shown with the reference ID (Ref. ID).

c) Induction ratio (IR) of CYP3A4 activity was calculated from daily dose and days of administration of rifampicin by using Eqs. 6–8 and 15–17. The values for IR were represented as an average and upper and lower limits when one S.D. for inter-individual variability of CYP3A4 baseline activity was considered.

For comparison, prediction using an indirect effect model was conducted. When the default *k_deg_* value of the Simcyp simulator (0.0072 h^–1^) and its corrected value for an *in vitro*-*in vivo* correlation (0.03 h^–1^) were used [51], [52], the *Q* ^2^ values were 0.499 and 0.570, respectively. In addition, the *Q* ^2^ values were estimated to be 0.604 when the prediction was made by a simple static model, where the average concentration of rifampicin in blood was calculated by dividing its *AUC* by the dosing interval (i.e., 24 h). The predictions provided by both cases were not as accurate as the presently proposed model.

### Simulation of non-steady state DDI using the PHML model

**Figure 7:**
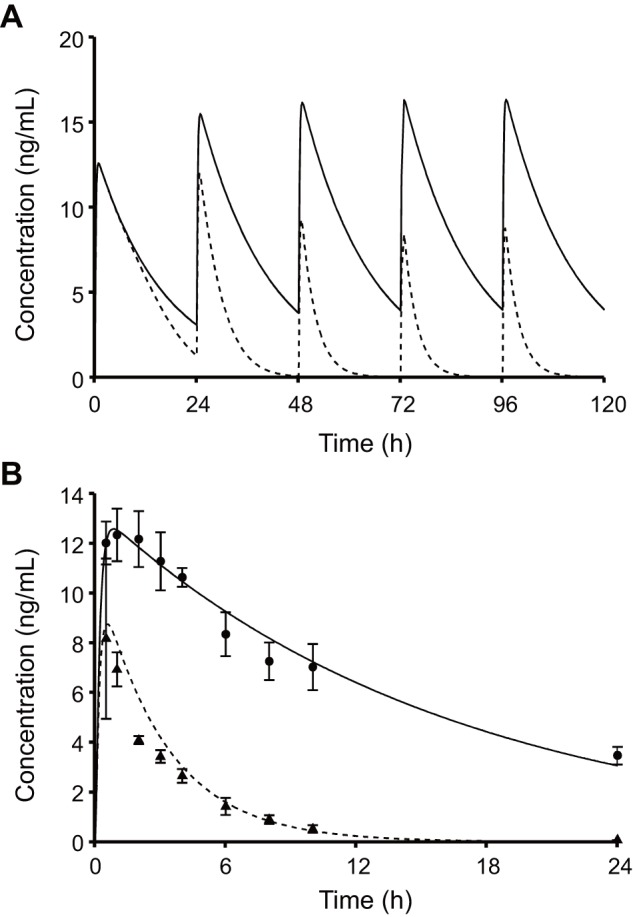
Simulation of DDI between alprazolam and rifampicin using a PHML/SBML hybrid model. Fig.(solid line) and presence (broken line) of rifampicin. Fig. 7B represents the comparison between the predicted blood concentration of alprazolam with the corresponding clinical data (Ref. 35). Keys: 1 mg alprazolam alone (•, solid line); 1 mg alprazolam with 4-day pretreatment with daily doses of 450 mg rifampicin (▴, broken line). The pharmacokinetic parameters for alprazolam were estimated by curve-fitting to the blood concentrations following the sole administration (standard deviation of residuals, *RSD*: 0.483), and then used for predicting those following the concomitant administration (standard deviation of prediction errors, *SDEP*: 0.760). Both *RSD* and *SDEP* was the same in terms of formula: 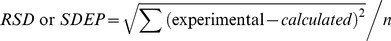.

Taking alprazolam as an example, of which the DDI was investigated under short-term treatment with rifampicin, the early phase of DDI was simulated. PBPK parameters for alprazolam (see Table S2) were obtained by curve-fitting to its blood concentration profile as has been reported previously [7], and PBPK models for alprazolam and rifampicin were implemented in PHML using PhysioDesigner. Fig. 7A shows simulations of the blood concentration of alprazolam in the presence and absence of co-administration of rifampicin. Both drugs were assumed to be administered orally every 24 h. In the absence of rifampicin, the blood concentration of alprazolam was increased stepwise following repeated oral doses and eventually reached a steady state. In contrast, in the presence of rifampicin the blood concentration of alprazolam decreased in a time-dependent manner and then reached a steady state at the lower level. Fig. 7B represents comparison between simulation results and measured clinical data [35]. The concentration profile of alprazolam with rifampicin treatment was predicted well (*SDEP*: 0.760), using pharmacokinetic parameters of both drugs and induction dynamics parameters for rifampicin. Pharmacokinetics of other drugs with relatively shorter-term rifampicin treatment were also simulated (see Figure S1), if the time-course data were available.

## Discussion

Rifampicin is a strong inducer of drug metabolizing enzymes such as CYP3A4. Rifampicin binds to the nuclear receptor pregnane X receptor (PXR). Once activated, PXR forms a heterodimer with the retinoic receptor (RXR), translocates into the nucleus, and acts as a transcriptional factor. Transactivation of PXR by rifampicin is regulated in a complex manner. Rifampicin-activated PXR is negatively regulated by the small heterodimer partner (SHP), which can be induced by farnesoid X receptor (FXR) ligands [53]. SHP was shown to prevent the PXR/RXR heterodimer from binding to DNA in a pull-down assay, while over-expression of SHP inhibited transactivation of PXR by rifampicin [53]. However, rifampicin-activated PXR is known to suppress expression of the SHP gene, while simultaneously interacting with HNF4α, SRC-1 and PGC-1α to initiate transcription of the CYP3A4 gene [54]. As shown in Fig. 2, the levels of CYP3A4 mRNA post administration of rifampicin (using data compiled from the literature), appear to be highest at around 48 h. In the present analysis, these observations were regarded as a consequence of gene expression regulatory networks and were described using a simplified negative feedback model.

It has been observed that upon repeated oral administration, the clearance of rifampicin increases because of self-induced metabolism [41], [47]. Since the enzyme responsible for the metabolism of rifampicin has recently been identified [55], it is still unclear whether its expression can be induced by a PXR-mediated mechanism, similar to CYP3A4 and other drug metabolizing enzymes [56]–[58]. Therefore, in order to construct a PBPK model for the analysis of rifampicin pharmacokinetics, a simple auto-induction process was considered. Based on the blood concentration profiles of rifampicin following repeated oral dosing, seven parameters for rifampicin were estimated. Simultaneous multiple curve-fitting allowed robust estimation of the pharmacokinetic parameters. Even the *K_p,h_* appeared to be reasonably estimated, despite the lack of hepatic distribution data. The *K_p,h_* obtained from the nonlinear regression analysis was 10.6, which fell within the range of *K_p,h_* values calculated from *in vivo* human biopsy data (4.8–30.3) [59]. This was also confirmed using the tissue composition-based equations reported by Poulin and Theil [60]. The *K_p,h_* for rifampicin was estimated at 6.01 using a computed octanol/water partition coefficient for rifampicin (logK_ow_: 4.24, obtained from EPI Suite, available at http://www.epa.gov/opptintr/exposure/pubs/episuite.htm).

*In vitro* parameters for rifampicin were estimated assuming that its degradation was negligible during the time period of the experiment. Even when the metabolism of rifampicin was incorporated into the *in vitro* CYP3A4 induction model using a reported generation rate of the metabolite [58], differences in parameter estimation were at most 16% (data not shown). A notable point of the analysis was that the parameter optimization procedure could be carried out directly without providing the *k_cyp,deg_* value. Because it was a parameter sensitive to the difference in the initial slope between the mRNA and activity profiles. In a conventional model which analyzes the activity profile alone, the maximally induced state needs to be presented in the profile to estimate the parameter. More interestingly, the *k_cyp,deg_* value estimated (0.0282 h^–1^) was rather close to 0.03 h^–1^, which was corrected for better *in vitro*/*in vivo* extrapolation [51], [52], than a default *k_deg_* value of the Simcyp simulator (0.0072 h^–1^). It has been reported that the turnover half-lives for CYP3A4 determined by various methods ranged from 10 to 140 h [61], which corresponds to 0.005–0.07 h^–1^. Although more information is needed to define an appropriate *k_deg_*, the reasonable estimate was obtained from the *in vitro* data.

The reduction of *AUC* because of rifampicin-induced DDI was satisfactorily predicted from *in vitro* CYP3A4 induction data (Fig. 5). The predictive correlation coefficient of the present dynamic model (*Q* ^2^: 0.684) was slightly better than that of a conventionally used indirect effect model with the *k_cyp,deg_* of 0.0072 h^–1^ (*Q* ^2^: 0.499) or 0.03 h^–1^ (*Q* ^2^: 0.570). Since these models can deal with the dynamics of CYP3A4 induction, the IRs for each drug were calculated according to the dosage regimen. As shown in Fig. 5, however, the level of CYP3A4 activity becomes stable on day 6 or later. Since most of the clinical DDI evaluations were carried out on these days (i.e. after 5 or more days of treatment with rifampicin), even a static model could also describe DDI (*Q* ^2^: 0.604). The advantage of dynamic models is that it allows the simulation of DDI even at the early stages of treatment. The present dynamic DDI model, which considers the induction of CYP at not only the activity level but also at the mRNA level, was shown to successfully simulate the clearance time-course of alprazolam, a drug known to be metabolized by CYP3A4 (Fig. 7).

Rifampicin is known to induce other CYP enzymes moderately, as has also been described in the FDA guidance [62]. When rifampicin is concomitantly administered, clearance of bupropion (a CYP2B6 substrate), repaglinide (a CYP2C8 substrate), and warfarin (a CYP2C9 substrate) increases 2.1∼3.4 times [63], 2.3 times [64], and 2.3∼3.8 times [65], [66], respectively. As compared with them, clearance of typical CYP3A4 substrates was much more induced (∼10 times) (Table 1). A review article [67] compiled information on DDI with rifampicin and indicated that rifampicin induces CYP3A4 more efficiently than other CYPs, glucuronosyltransferases (UGTs), and p-glycoprotein. Taking them into account, induction of other enzymes than CYP3A4 would minimally affect the results of prediction, unless the *fm_CYP3A4_* of substrates was extremely low. Gefitinib (*fm_CYP3A4_*: 0.39) is known to be metabolized largely by CYP2D6 [68], which is little induced by rifampicin. On the other hand, imatinib (*fm_CYP3A4_*: 0.28) is metabolized by CYP2C8 to the similar extent with CYP3A4 [69], resulting in slightly possible underestimation of DDI due to rifampicin. Prednisolone (*fm_CYP3A4_*: 0.18) has been reported not to be metabolized by any other CYPs than CYP3A4 or UGTs [70]. Although the reasons why the *fm_CYP3A4_* of prednisolone is low remain unclear, the *fm_CYP3A4_* of 0.18 gave a good prediction of the DDI due to rifampicin. As long as the results were viewed as fair, induction of other enzymes or transporters might not be important in determining DDI between CYP3A substrates and rifampicin.

PHML, which inherited *insilico*ML (ISML) [71], is a new XML-based specification to describe a wide variety of models of biological and physiological functions with hierarchical structures. It can describe mathematical models consisting of ordinary differential equations, partial differential equations, agent-based simulation models, and others. In a similar way to ISML [71], a model is described by a set of functional elements (modules), each of which specifies mathematical expressions of the module functions. PhysioDesigner acts as a graphical editor and browser of the models written in PHML or ISML. A notable feature of PhysioDesigner is that it provides a function for creating SBML-PHML hybrid models. Since SBML is widely distributed as a standard format for representing and sharing models of biochemical reaction networks, it enables us to create multi-level physiological model systems. The functions of PhysioDesigner allowed us to dynamically connect PBPK-based DDI models with an enzyme transcription/translation dynamics model. Since the module-based hybrid model is highly reusable, extension to more comprehensive network models would be expected in future.

## Supporting Information

Figure S1 **Simulation of blood concentration of CYP3A4 substrate drugs following their oral administration.** Keys: sole administration (•, solid line); 5-day pretreatment with daily doses with 600 mg rifampicin (▴, dash line). Pharmacokinetic parameters for each drug were estimated by curve-fitting to the blood concentrations following the sole administration, and then used for predicting those following co-administration with rifampicin. The pharmacokinetic parameters are given in Table S2.
(DOC)

Table S1 **Pharmacokinetic data for CYP3A4 substrates.**
(DOC)

Table S2 **Pharmacokinetic parameters of CYP3A4 substrates.**
(DOC)
