# Supplementary material for: Modeling of Rifampicin-Induced CYP3A4 Activation Dynamics for the Prediction of Clinical Drug-Drug Interactions from In Vitro Data
Source: PLoS One. 2013 Sep 24;8(9):e70330. doi: 10.1371/journal.pone.0070330 (PMC3782498; doi:10.1371/journal.pone.0070330)
Supplement: Figure S1 — Simulation of blood concentration of CYP3A4 substrate drugs following their oral administration. Keys: sole administration (•, solid line); 5-day pretreatment with daily doses with 600 mg rifampicin (▴, dash line). Pharmacokinetic parameters for each drug were estimated by curve-fitting to the blood concentrations following the sole administration, and then used for predicting those following co-administration with rifampicin. The pharmacokinetic parameters are given in Table S2. (DOC) [file pone.0070330.s001.doc]

**Figure S1.** Simulation of blood concentration of CYP3A4 substrate drugs following their oral administration. Keys: sole administration (●, solid line); 5-day pretreatment with daily doses with 600 mg rifampicin (▲, dash line). Pharmacokinetic parameters for each drug were estimated by curve-fitting to the blood concentrations following the sole administration, and then used for predicting those following co-administration with rifampicin. The pharmacokinetic parameters are given in Table S2.
