# Supplementary material for: Modeling of Rifampicin-Induced CYP3A4 Activation Dynamics for the Prediction of Clinical Drug-Drug Interactions from In Vitro Data
Source: PLoS One. 2013 Sep 24;8(9):e70330. doi: 10.1371/journal.pone.0070330 (PMC3782498; doi:10.1371/journal.pone.0070330)
Supplement: Table S1 — Pharmacokinetic data for CYP3A4 substrates. (DOC) [file pone.0070330.s002.doc]

**Table S1.** Pharmacokinetic data for CYP3A4 substrates

| Substrate | Subject information | | |  | Pharmacokinetic data*^a^* | | | | | Ref. ID |
| --- | --- | --- | --- | --- | --- | --- | --- | --- | --- | --- |
|  | number | age | weight  (kg) |  | rifampicin | AUC_0→∞_  (ng·h/mL) | C_max_  (ng/mL) | t_max_  (h) | t_1/2_  (h) |  |
| alprazolam | 4 | — | — |  | – | 242.2±37.9 | 15.2±1.5 | 1.2±0.4 | 14.1±1.4 | 35 |
|  |  |  |  |  | 450mg, 4days | 28.4±3.4 | 9.7±2.6 | 0.9±0.1 | 2.6±0.3 |  |
| atorvastatin | 10  (6M4F) | 18–31 | 55–78 |  | – | 64.0±21.3 | 15.8±5.4 | 0.5  (0.5–1.5) | 10.3±1.2 | 25 |
|  |  |  |  |  | 600mg, 5days | 12.6±3.2 | 9.5±3.7 | 0.5  (0.5–1) | 2.7±0.9 |  |
| buspirone | 6  (2M4F) | 18–24 | 60–85 |  | – | 18.7±11.6 | 5.2±2.8 | — | — | 28 |
|  |  |  |  |  | 600mg, 5days | 1.65±0.36 | 0.76±0.23 | — | — |  |
| cyclosporine | 6  (5M1F) | 26–46 | 57–100 |  | – | 8996±3813 | — | — | — | 36 |
|  |  |  |  |  | 600mg, 11days | 2399±1014 | — | — | — |  |
| gefitinib | 18  (18M) | 27–56 | 59–96 |  | – | 5044  (CV: 88%) | 167.5  (CV: 53%) | 3.0  (3.0–7.0) | 34±14 | 31 |
|  |  |  |  |  | 600mg, 16days | 840  (CV: 71%) | 58.8  (CV: 68%) | 3.0  (1.0–5.0) | 21±8 |  |
| imatinib | 14  (13M1F) | 40–64 | 61.5–90 |  | – | 22992±5607 | 1563±285 | 2.5  (2.0–4.0) | 16.7±3.1 | 26 |
|  |  |  |  |  | 600mg, 11days | 5996±1631 | 727±173 | 2.5  (1.0–2.5) | 8.8±0.7 |  |
| mefloquine | 7  (7M) | 24–35 | 52–72 |  | – | 373700±57500 | 855.6±168.0 | 8.2±2.9 | 305.3±47.2 | 38 |
|  |  |  |  |  | 600mg, 7days | 119800±54900 | 695.7±56.6 | 8.7±3.9 | 113.4±49.7 |  |
| midazolam | 6  (4M2F) | 19–30 | 50–93 |  | – | 170±13.3* | 55±4* | 1  (1–3) | 3.1±0.2* | 40 |
|  |  |  |  |  | 600mg, 5days | 7.00±0.83* | 3.5±0.7* | 1.25  (1–2) | 1.3±0.2* |  |
| midazolam | 19  (9M10F) | 38.7±8.8 | 73.4±11.3 |  | – | 49  (22–103) | 23  (9–41) | 0.5  (0.5–1) | 1.63  (1.22–2.75) | 27 |
|  |  |  |  |  | 600mg, 9days | 6.1  (3.4–10) | 3.9  (1.7–6.2) | 0.27  (0.2–1) | 0.93  (0.75–1.10) |  |
| nifedipine | 6  (4M2F) | 25±2 | 73±9 |  | – | 229.9±33.8 | — | — | 2.3±1.4 (i.v.) | 37 |
|  |  |  |  |  | 600mg, 7days | 18.8±8.6 | — | — | 1.7±1.0 (i.v.) |  |
| prednisolone | 7 | 16–65 | 45–75 |  | – | 2699±1015 | 536.4±188 | — | 3.58±1.26 | 30 |
|  |  |  |  |  | 480mg, 30days | 1333±169 | 419.4±110 | — | 3.03±0.89 |  |
| simvastatin | 10  (10M) | 21–29 | 59–81 |  | – | 17.3±9.9 | 5.6±2.2 | 1  (0.5–1.5) | 3.2±1.1 | 27 |
|  |  |  |  |  | 600mg, 9days | 2.40±1.81 | 0.6±0.5 | 1  (0.5–6) | 3.2±2.2 |  |
| simvastatin | 19  (9M10F) | 38.7±8.8 | 73.4±11.3 |  | – | 29  (8–56) | 7.4  (3.1–19) | 1  (0.5–9) | 2.92  (0.75–8.95) | 29 |
|  |  |  |  |  | 600mg, 5days | 2.6  (0.8–26) | 1.1  (0.3–7.2) | 1  (0.5–6) | 1.93  (0.60–14.3) |  |
| telithromycin | — | — | — |  | – | — | — | — | — | 39 |
|  |  |  |  |  | 600mg, 7days | 14% of control  (9–31%) | 21% of control  (10–37%) | — | — |  |
| triazolam | 10  (6M4F) | 19–25 | 53–90 |  | – | 14.8±1.0* | 2.9±0.2* | 1.5  (1–3) | 2.8±0.1* | 34 |
|  |  |  |  |  | 600mg, 5days | 0.74±0.14* | 0.36±0.06* | 1.5  (0.5–2) | 1.3±0.1* |  |
| zolpidem | 8  (8F) | 21–25 | 54–68 |  | – | 1202±157* | 293±61* | 1.5  (0.5–2) | 2.5±0.2* | 33 |
|  |  |  |  |  | 600mg, 5days | 336±67* | 117±25* | 1.75  (1–3) | 1.6±0.1* |  |
| zopiclone | 8  (5M3F) | 22–24 | 55–84 |  | – | 473±114 | 76.9±27.2 | 3  (0.5–4) | 3.8±0.6 | 32 |
|  |  |  |  |  | 600mg, 5days | 86.1±34.5 | 22.5±6.0 | 1.25  (0.5–3) | 2.3±0.9 |  |

a) Pharmacokinetic data are basically represented as mean ± S.D, unless marked with asterisks (mean ± S.E.M.).
