# Supplementary material for: Modeling of Rifampicin-Induced CYP3A4 Activation Dynamics for the Prediction of Clinical Drug-Drug Interactions from In Vitro Data
Source: PLoS One. 2013 Sep 24;8(9):e70330. doi: 10.1371/journal.pone.0070330 (PMC3782498; doi:10.1371/journal.pone.0070330)
Supplement: Table S2 — Pharmacokinetic parameters of CYP3A4 substrates. (DOC) [file pone.0070330.s003.doc]

**Table S2.** Pharmacokinetic parameters of CYP3A4 substrates

|  | K_p,h_*^a^* | F_a_F_g_*^a^* | CL_r_*^a^*  (L/h) | fu_p_*^a^* | R_b_*^a^* | k_a_*^b^*  (h^–1^) | V_1_*^b^*  (L) | k_12_*^b^*  (h^–1^) | k_21_*^b^*  (h^–1^) | CL_int,h_*^b^*  (L/h) | fm_CYP3A4_*^c^* |
| --- | --- | --- | --- | --- | --- | --- | --- | --- | --- | --- | --- |
| alprazolam | 2.29 | 0.901 | 0.575 | 0.29 | 0.81*^d^* | 5.77 | 76.8 |  |  | 12.8 | 0.75 |
| atorvastatin | 0.73 | 0.141 | 0.298 | 0.02 | 1 | 1.50 | 41.8 | 3.27 | 0.448 | 4500 | 0.68 |
| midazolam | 6.59 | 0.615 | 0.00801 | 0.05 | 0.675 | 0.739 | 64.1 |  |  | 1210 | 0.92 |
| nifedipine | 0.75 | 0.823 | 0.0309 | 0.044 | 0.848 | 1.12 | 42.2 |  |  | 1810 | 0.78 |
| simvastatin | 6.91 | 0.077 | 0 | 0.06 | 1 | 0.723 | 88.4 |  |  | 3230 | 1.00 |
| triazolam | 7.26 | 0.518 | 0.263 | 0.099 | 1 | 0.662 | 36.7 |  |  | 181 | 0.93 |
| zolpidem | 5.84 | 0.886 | 0.183 | 0.08 | 0.66 | 0.580 | 7.48 | 2.17 | 1.08 | 199 | 0.40 |
| zopiclone | 0.99*^e^* | 0.912*^f^* | 0.562*^f^* | 0.45*^f^* | 0.89*^d^* | 0.271 | 40.1 |  |  | 46.0 | 0.44 |

1. The values were obtained from the article of Kato, et al. (Ref. 8) unless described otherwise.
2. The values were estimated by curve-fitting to the blood concentration profile, according to the method described in Ref. 8
3. The values were obtained from the article of Ohno, et a. (Ref. 23).
4. The values were obtained from the article of Jantos, et al. (Arch Kriminol 227: 188–203, 2011).
5. The value was derived from the computed octanol/water partition coefficient (logK_ow_: 1.54) of zopiclone, according to the method described in Ref. 8.
6. The values were obtained from the article of Fernandez, et al. (Clin Pharmacokinet 29: 431–441, 1995).
